# Moderate Partially Reduplicated Conditioned Stimuli as Retrieval Cue Can Increase Effect on Preventing Relapse of Fear to Compound Stimuli

**DOI:** 10.3389/fnhum.2017.00575

**Published:** 2017-11-30

**Authors:** Junjiao Li, Wei Chen, Jingwen Caoyang, Wenli Wu, Jing Jie, Liang Xu, Xifu Zheng

**Affiliations:** ^1^School of Psychology, South China Normal University, Guangzhou, China; ^2^Center for Studies of Psychological Application, South China Normal University, Guangzhou, China; ^3^Guangdong Key Laboratory of Mental Health and Cognitive Science, South China Normal University, Guangzhou, China; ^4^School of Marxism, South China University of Technology, Guangzhou, China

**Keywords:** fear, retrieval-extinction, memory reconsolidation, compound stimuli, boundary condition, retrieval ratio

## Abstract

The theory of memory reconsolidation argues that consolidated memory is not unchangeable. Once a memory is reactivated it may go back into an unstable state and need new protein synthesis to be consolidated again, which is called “memory reconsolidation”. Boundary studies have shown that interfering with reconsolidation through pharmacologic or behavioral intervention can lead to the updating of the initial memory, for example, erasing undesired memories. Behavioral procedures based on memory reconsolidation interference have been shown to be an effective way to inhibit fear memory relapse after extinction. However, the effectiveness of retrieval–extinction differs by subtle differences in the protocol of the reactivation session. This represents a challenge with regard to finding an optimal operational model to facilitate its clinical use for patients suffering from pathogenic memories such as those associated with post-traumatic stress disorder. Most of the laboratory models for fear learning have used a single conditioned stimulus (CS) paired with an unconditioned stimulus (US). This has simplified the real situation of traumatic events to an excessive degree, and thus, limits the clinical application of the findings based on these models. Here, we used a basic visual compound CS model as the CS to ascertain whether partial repetition of the compound CSs in conditioning can reactivate memory into reconsolidation. The results showed that the no retrieval group or the 1/3 ratio retrieval group failed to open the memory reconsolidation time window. The 2/3 repetition retrieval group and the whole repetition retrieval group were able to prevent fear reinstatement, whereas only a 2/3 ratio repetition of the initial compound CS as a reminder could inhibit spontaneous recovery. We inferred that a retrieval–extinction paradigm was also effective in a more complex model of fear if a sufficient prediction error (PE) could be generated in the reactivation period. In addition, in order to achieve an optimal effect, a CS of moderate discrepancy should be used as a reminder.

## Introduction

Memory is not an exact copy but rather an adaptive presentation of a past experience. The dynamic nature of memory provides opportunities for people to change the content, strength or even emotional valence of memories to events (Nader et al., 2013). In pre-clinical studies of anxiety disorders, classical Pavlovian fear conditioning is used as the basic model of fear learning, which has a similar mechanism to anxiety disorders and post-traumatic stress disorders in humans (Jones and Monfils, 2016). The acquired fear can be eliminated gradually if the conditioned stimulus (CS) is no longer followed by an unconditioned stimulus (US) such as electric shock, which is the principle of “exposure therapy”. However, fears extinguished successfully are prone to recover if the patient encounters the US after extinction (“reinstatement”), returned to the original context of conditioning (“renewal”), or as a result of the passage of time (“spontaneous recovery”; Bouton, 2002). The standard extinction forms a new inhibitory memory which competes with the acquired fear memory (Rothbaum and Davis, 2003; Jones and Monfils, 2016), the behavioral response to fear being the result of competition, which leaves the original fear memory intact. How to eliminate the acquired fear memory thoroughly and prevent relapse has been the focus of several scholars.

In recent years, a series of studies based on “memory reconsolidation interference” has helped to resolve this problem. The theory of memory reconsolidation states that, if a consolidated memory is recalled or reactivated, it will return to a fragile and unstable state and need stabilization to become steady again. This process also requires protein synthesis and lasts ≈6 h (the “reconsolidation time window”; Nader et al., 2000). Hence, in recent years, researchers have tried to find ways that use this period to prevent fear memories becoming stable again or to erase maladaptive memories.

Studies have suggested that use of the β-adrenergic receptor antagonist propranolol or certain behavioral interventions during the reconsolidation period enable memory to be “updated” or “rewritten” (Sandrini et al., 2015). In drug-intervention studies, Kindt et al. (2009) were the first to demonstrate the amnestic effect of propranolol on a reactivated fear memory in humans. In studies on behavioral intervention, a new training paradigm was proposed by Monfils et al. (2009). It was shown that undertaking “extinction training” during the reconsolidation window could eliminate the fear memory in rats because a relapse on spontaneous recovery or reinstatement tests was not observed. In 2010, Schiller translated this behavioral paradigm, which is called “retrieval–extinction”, to humans and showed its effectiveness in preventing fear relapse that could last 12 months (Schiller et al., 2010). A classical retrieval–extinction paradigm is a 3-day model comprising fear acquisition on day-1, memory retrieval and fear extinction on day-2 and test of fear recovery on day-3. On day-1, participants acquire the aversive valance of the CS+ through a Pavlovian fear-conditioning procedure. After 24 h, they return to the former environment and their fear memory is reactivated by a presentation of CS+ only once, without an US. After 10 min (during the reconsolidation window), a standard extinction process is executed. Then, 24-h later, a test of spontaneous recovery or reinstatement is taken to ascertain if the fear memory was extinguished on day-2.

This new training paradigm opens up the possibility of using these strategies to treat memory disorders, and has drawn research attention recently. One must show that interruption of memory reconsolidation can prevent re-stabilization of the retrieved memory as well erasure of the conditioned behavioral response, as assessed in follow-up tests, in the aversive memory and appetitive memory (Kindt et al., 2009; Agren et al., 2012; Xue et al., 2012; Liu et al., 2014; Zeng et al., 2014).

It has been argued that interference of memory reconsolidation produces a modification/update of the original memory rather than forming new extinction learning. The mechanism underlying the process is based on synaptic plasticity and shows the dynamic nature of memory (Bonin and De Koninck, 2015; Nader, 2015). The neural mechanism of retrieval extinction is different to that of traditional extinction. That is, the prefrontal cortex is engaged in standard extinction but not in extinction during reconsolidation (Schiller et al., 2013; Bjorkstrand et al., 2015).

Some studies have failed to show the effectiveness of the behavioral memory updating paradigm (Chan et al., 2010; Ishii et al., 2015). For instance, some human studies have failed to replicate the findings of the retrieval–extinction procedure (Soeter and Kindt, 2011; Golkar et al., 2012; Kindt and Soeter, 2013; Meir Drexler et al., 2014; Schroyens et al., 2017). The observed inconsistencies could be the result of the absence of an agreed or standard behavioral parameter (Nader, 2015). There is mounting evidence that the reconsolidation impairment paradigm works only if the memory and operation meet certain conditions; and specific conditions under which memory can be destabilized are called “boundary conditions”.

Several boundary conditions have been proposed: memory age, training strength, duration of retrieval, trace dominance and the number of retrieval trials (Eisenberg et al., 2003; Suzuki et al., 2004; Wang et al., 2009; Winters et al., 2009; Robinson and Franklin, 2010; de Beukelaar et al., 2014). More recently, the prediction error (PE) has been suggested to be a boundary condition of memory reconsolidation (Winters et al., 2009; Diaz-Mataix et al., 2013; Sevenster et al., 2013, 2014). The PE is defined as a mismatch between what is expected and what really occurs. The PE has been demonstrated to be a necessary condition to destabilize the memory and open the reconsolidation window (Exton-McGuinness et al., 2015). However, consensus on the criteria of behavioral operations for testing of these boundary conditions is lacking.

Various studies in humans and animals have explored the mechanisms of retrieval–extinction at behavioral, system and molecular levels, but the memory model used by most studies were single cue CS paired with US conditioning model, in which the CS just had one cue. For example, Schiller et al. (2010) used a blue square or yellow circle as the CS that followed a mild electric shock or did not follow a mild electric shock, respectively. If there was more than one CS, each CS was single-paired with an US and the compound CSs were presented successively rather than simultaneously. This model is effective for exploring the nature of fear memory, but it cannot answer the question that if there is more than one memory cue, or whether partial repetition of the whole used as reminders can reactivate the memory to undergo reconsolidation.

A traumatic event contains many related cues but, in clinical therapy, all of the cues cannot be used to retrieve memories. Hence, selection of an effective CS as the indicator and finding an optimal paradigm to reactivate memories is crucial for the translation of this promising procedure into clinical use. Jones et al. (2013) used multiple stimuli as the CS in rats to ascertain if the effect of post-retrieval extinction paradigm in more complex fear model is similar to a simple model of fear. They used a tone and a light (T + L) presented simultaneously as the CSs+ followed by a shock to build the associative learning. Then they compared the extinction characteristics between complex fear memory and simple tone-shock pairings and explored the paradigm to destabilize and distinguish fear of complex cues. They showed that fear to compound CSs is more resistant to extinction than simple fear pairing. And the effect of retrieval extinction in complex fear model is dependent upon a particular extinction paradigm (Jones et al., 2013). However, few studies have used compound CSs as the CS in healthy adults. During the reactivation session, the number of parts of the compound CSs that need to be exposed to open the reconsolidation window is not known.

In the present study, we used a basic visual multiple-stimuli model as the CS to explore the possible factors that determine the efficiency of the post-reactivation extinction paradigm. This CS model contained three solid figures in different colors presented simultaneously and which were followed by a mild electric shock to the wrist (CS+) or not (CS−). The next day, none, one, two, or all three elements of the initial CSs were used as retrieval cues and were followed by traditional extinction training 10 min after retrieval. The different effects on inhibition of fear relapse were tested on day-3.

In the error-correction models of associative learning (Rescorla and Wagner, 1972), omission of the expected US produces an error that influences the subsequent associability of the CS. In the Rescorla–Wagner model, an increase or decrease from the expectancy strength of the US to the actual US elicits excitatory or inhibitory learning of the CS. However, in the Pearce–Hall model, either condition elicits an increase in the associability of the CS (i.e., the ability to enter into new associations). Hence, if the PE is large, the associability of the CS is high, which facilitates later associative learning of the CS or a change in the initial CS–US connection. In the same way, if the PE is small, the associability of the CS is low, and will not change former learning into safe associativity or build new learning.

Based on rate-based models of conditioning (Gallistel and Gibbon, 2000), the content of learning of an individual is a linear function of r, the reinforcement rate of the CS. Hence, if ABC compound CSs are paired with a reinforcement rate rABC, then rAB = rABC − rC. So when AB were presented, participants will expect 2/3 amount of electronics. The absence of an electric shock will then produce a lower PE than appearance of the entire ABC compound.

In the present study, the conditioning formed a connection between a three-compound CS with a US with a reinforcement rate of 60%. Hence, one trial of presentation of a single CS (as in a 1/3 CS repetition retrieval) on day-2 would produce the least PE in the three groups. As stated above, a too-low PE will fail to produce sufficient associability of the CS to enter into a new connection, which inhibits extinction training. However, a 3/3 CS repetition can elicit the largest PE, which corresponds to the highest level of CS associability in the three groups. In the 2/3 repetition retrieval group, the expectancy of the US is US = 2/3r, which could produce sufficient associability of the CS and change the original CS–US connection. Some researchers have argued that a large PE can promote learning by engaging the attention mechanisms that contribute to the establishment of long-term memories (Janak et al., 2012). We speculate that a change in the CS can attract more attention from the participant or stimulate more arousal, which is needed to strengthen reconsolidation. Hence, 2/3 repetition retrieval may have better effects than whole retrieval of the CS.

Therefore, the hypothesis of the present study was that, if participants acquire three elements of fear conditioning on the compound CS on day-1, using a greater proportion of replication (i.e., two or three elements) as retrieval cues can effectively reactivate fear memory into reconsolidation and then modify the fear memory into safe memory through a behavioral-interference procedure. However, if using fewer elements (i.e., none or one), retrieval cannot destabilize the former memory so fear memory will emerge after extinction.

## Materials and Methods

### Participants

All participants provided written informed consent in accordance with the Declaration of Helsinki and were paid for their participation. The participants who had completed the experiments were paid a small amount of money (RMB 50 + traffic cost, i.e., RMB12) at the end of the third day. For participants who failed to acquire conditioned fear and cannot continue the following experiments, they were given RMB 15 at the end of the first day for their participation. The research had been approved by the human research ethics committee for non-clinical faculties of School of Psychology, South China Normal University (Approval Number: 117).

Ninety-two (33 male; 59 female) healthy individuals were recruited by advertisements (posters placed in colleges; Internet). Most participants were college students aged 18–35 years. Participants were placed randomly into three experimental groups and a control group. Participants were excluded from statistical analyses if they did not acquire fear conditioning or extinguish fear conditioning on day-1 and day-2, respectively, as assessed by a skin conductance response (SCR). This criterion was based on the mean differential skin conductance responses (md SCR) to CS+ and CS− on the last half of conditioning trials or extinction trials. According to Schiller et al. (2010) the specific criteria are that the difference during acquisition should not go in the opposite direction (CS− > CS+) or <0.1 μs; and during extinction, the difference should not go in the opposite direction (CS+ > CS−) or >0.1 μs. 12 participants (3 from the 1/3 retrieval group, 2 from the 2/3 retrieval group, 3 from the whole retrieval group, and 4 from the no retrieval group) were excluded according to these criteria.

Hence, participants after excluded were 20 persons in each of the 1/3 repetition retrieval group (8 males), 2/3 repetition retrieval group (8 males), 3/3 repetition retrieval group (8 males) and no retrieval group (9 males). A description separated by groups of participants with information of sex is shown in Supplementary Table S1 (Supplementary Materials).

### Stimuli

**Figure 1:**
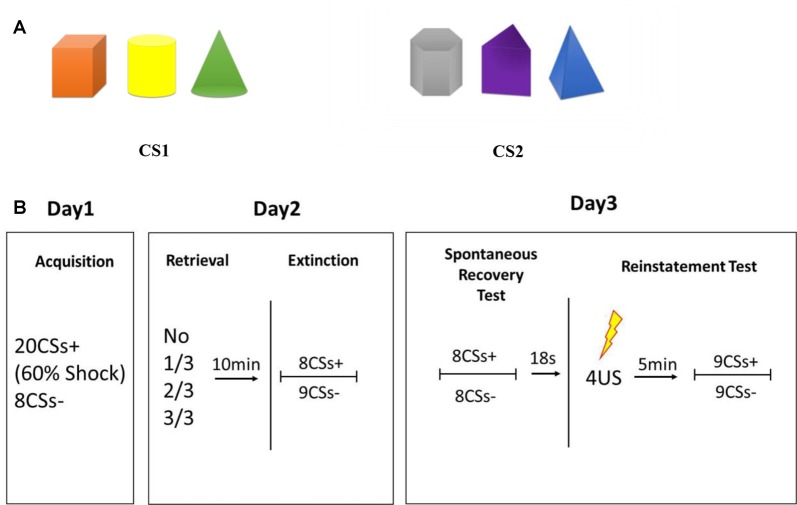
**(A)** Conditioned stimulus (CS). **(B)** Schematic representation of the experimental design. During acquisition, 20 trials of CS were presented to participants with a reinforcement ratio of 60%, which were: 12 CS+ accompanied by an electric shock; 8 CS+ without an electric shock; 8 CS− without an electric shock. During extinction, 8 CS+ and 9 CS− were presented, all with no shock. The first CS− trial in each phase was disregarded due to the orientation response at the beginning of the session.

The CS comprised two types of pictures, each of which consisted of three solid figures of different shapes and colors (Figure 1A). The position of the three figures was counterbalanced with each other, and each figure was chosen randomly as a reminder cue in experimental groups. Colors were randomized among different conditions and six kinds of sequences were used to balance the color or shape that used as CS+ and retrieval cues (see Supplementary Figure S1). The CS was presented for 5 s in a pseudo-randomized, interspersed manner, the last 200 ms of which was accompanied with a US on 60% of CS+. The US consisted of an electrical stimulus given for 200 ms with a current of 50 pulses/s, which was determined individually to be “uncomfortable but not painful” at the beginning of the experiment. “Mild” electric shocks were delivered through a stimulating bar electrode attached to the wrist of the right hand. A conductive gel was used between the skin and electrodes. Shock deliverance was controlled by a constant-current stimulator. Inter-trial intervals (ITIs) varied from 8 s to 10 s.

### SCR

SCR was measured using Spirit NeXus-10 (BioTrace Medical, San Carlos, CA, USA) and the signal was sampled at 120 Hz. SCR is one of the most commonly used measurement of fear responding in humans (Lonsdorf et al., 2017). Electrodes were attached to the first and second fingers of the left hand, between the first and second phalanges. SCR waveforms were analyzed offline using BioTrace+ software for NeXus-10. SCR amplitudes to the CS and US were the dependent measures of conditioned and unconditioned responses, respectively. The level of the SCR response was determined by taking the base-to-peak difference for the first waveform in the 0.5–5.5-s window after stimulus onset. We used the criteria that the minimal response should above 0.02 μs. And responses did not pass the criterion were scored as zero (Schiller et al., 2010; Golkar et al., 2012). Raw SCR scores were square root-transformed to normalize distributions. These normalized scores were scaled according to the unconditioned response of each participant by dividing each response by the mean square root-transformed US response. This is a well-established approach for the examination of electro-dermal reactivity proposed by Schiller et al. (2010) and has been demonstrated in human psychophysiological research.

### Behavioral Tests

The entire experiment comprised three successive days. All participants had to attend the experiment at the same time on the 3 days. All behavioral procedures are illustrated in Figure 1B.

#### Fear Conditioning

Participants sat in front of a personal computer with the SCR and shock-electrode devices attached to their fingers and wrists. Before conditioning learning, evaluation of electric-shock strength was done to choose the uncomfortable but not painful shock strength for each participant. During conditioning training, 20 trials of a CS were presented to participants with a reinforcement ratio of 60%, which were 12 CS+ accompanied by an electric shock, eight CS+ without an electric shock, and eight CS− without an electric shock. CS trials appeared in a pseudo-randomized order with the criterion that the first trial is not followed by shock; no more than 2 CS trials are of the same type; and no more than 2 concessive stimuli are followed by shock. And assignment of the pictures as CS+ was counterbalanced across participants. Participants were trained to respond to two discriminable types of visual stimuli: a picture containing three “cold colored” solid figures (blue triangle cone, gray hexahedron, purple trihedron) or “warm colored” solid figures (orange cube, yellow cylinder, green cone) to form a distinct conditioned fear to CS+.

#### Reactivation Sessions and Extinction

Twenty-four-hours later, participants who acquired fear conditioning were divided randomly into four groups that differed in the number of CS components as retrieval cues: one figure, two figures, all three figures and no retrieval. During the reactivation session, an unreinforced retrieval cue was presented for 5 s to participants, and then they were given a 10-min break during which they watched an excerpt from a documentary from the British Broadcasting Corporation called *Planet Earth*. After watching, extinction training was executed by eight presentations of CS+ and nine of CS−, which was not followed by an electric shock. The first CS− trial in each phase was disregarded due to the orientation response at the beginning of the session (Schiller et al., 2010).

#### Re-Extinction and Reinstatement Test

Twenty-four-hours after post-retrieval extinction, participants took part in tests of spontaneous recovery and reinstatement of fear through re-extinction and regaining. During re-extinction, non-reinforced presentations of eight trials of CS+ and eight trials of CS− were presented randomly. Eighteen seconds after re-extinction, four un-signaled electric shocks were administered to the wrist without any instruction or presentation of stimuli. The ISI of four un-signaled shocks was 1 s. Then, a 5-min break was taken followed by nine unreinforced CS+ and nine CS− trials to test reinstatement of fear. The first CS+ and CS− trials were disregarded due to the orientation response at the beginning of the session (Schiller et al., 2010). During all sessions of the experiment, the electric-shock stimulator was set to the “on” position and the SCR was recorded continuously.

### Statistical Analyses

We used non-reinforced trials of CS+ and CS− in the analysis to exclude the impact of the electric shocks themselves on behavioral responses. The main dependent variable was the md SCR, which was calculated by subtracting responses to the CS− from those to the CS+ in each trial, and then averaging across participants. The md SCR was subjected to a mixed analysis of variance (ANOVA) for repeated measures between factors of groups (1/3 repetition retrieval, 2/3 repetition retrieval, whole retrieval and no retrieval) and within-subject factors of time (early and late phases).

Acquisition of fear was assessed by paired *t*-tests of the differential responses in the last half of the acquisition in each group; and extinction was assessed by comparing the differential responses of the last trial of extinction. To test spontaneous recovery, we compared the md SCR of the first re-extinction trial with that of the last extinction trial. Then, one-way ANOVA between the four groups of differential response of the first trial on day-3 was taken to compare the relative superiority for preventing spontaneous recovery in each group.

To test fear reinstatement, we compared the md SCR of the first trial of final extinction with that of the last re-extinction trial. Then, a one-way ANOVA test between the differential responses of the four groups to CS+ and CS− of the first trial of the reinstatement test was carried out to explore the comparable superiority for prevention of fear reinstatement.

*Post hoc* tests were performed using Fisher’s LSD between groups. We adopted a significant level of 0.05 and report partial *η*^2^ as the estimate of effect size. Greenhouse-Geisser adjustments of degrees of freedom were used when appropriate.

## Results

### Acquisition of Conditioned Fear Memory

**Figure 2:**
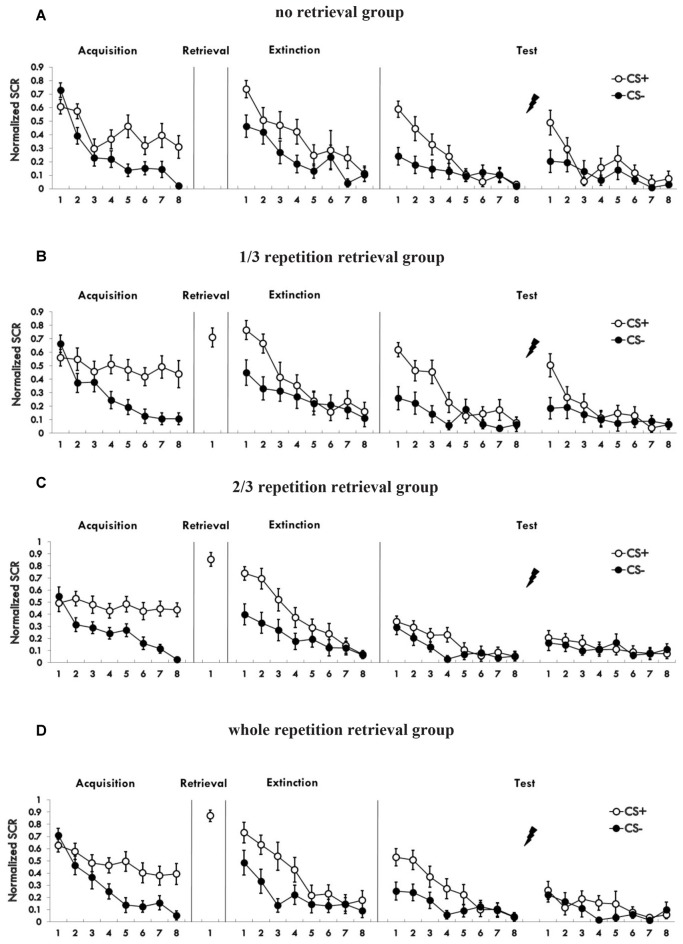
**(A–D)** Mean skin conductance responses (SCRs) to the CS+ and CS− trials during acquisition, reactivation, extinction and spontaneous recovery test and reinstatement test in each group.

Figure 2 shows the mean skin conductance responses to the CS+ and CS− trials during acquisition (Supplementary Figure S2). A mixed ANOVA with a between-subject factor of groups and within-factor of the training stage and stimulus type revealed a significant main effect of the training stage (*F*_(1,76)_ = 101.974, *p* < 0.01, *η*^2^ = 0.573) and the stimulus type (*F*_(1,76)_ = 90.662, *p* < 0.01, *η*^2^ = 0.544), but no significant effect of the groups (*F*_(3,76)_ = 0.333, *P* = 0.801 (i.e., >0.05), *η*^2^ = 0.013). There was a significant interaction between the stimulus type × training stage (*F*_(1,76)_ = 33.142, *p* < 0.01, *η*^2^ = 0.304), but no significant interaction between the group × training stage (*F*_(3,76)_ = 0.961, *P* = 0.416 (i.e., >0.05), *η*^2^ = 0.037) and the group × stimulus type (*F*_(3,76)_ = 0.368, *P* = 0.776 (i.e., >0.05), *η*^2^ = 0.014). There was also no significant interaction of the three factors (*F*_(3,76)_ = 0.24, *P* = 0.868 (i.e., >0.05), *η*^2^ = 0.009). A paired *t*-test of the last four trials of the training session in each group showed a significant higher SCR to CS+ than to CS− (no retrieval: *t* = 3.983, *p* < 0.01; 1/3 group: *t* = 5.342, *p* < 0.01; 2/3 group: *t* = 8.433, *p* < 0.01; 3/3 group: *t* = 3.732, *p* < 0.01). One-way analysis of the md SCR of the last four trials revealed no significant differences in different groups (*F*_(3,76)_ = 0.262, *P* = 0.853 (i.e., >0.05), *η*^2^ = 0.01). These results showed that participants acquired a conditioned fear to the CS+ but not to the CS−, and that the fear memory acquired by each group was of the same strength.

### Post-Retrieval Extinction

Figure 2 shows the SCR to CS+ and CS− trials during post-retrieval extinction phase. A mixed ANOVA with groups and training stage and stimulus type revealed a significant main effect of the training stage (*F*_(1,76)_ = 100.229, *p* < 0.01, *η*^2^ = 0.569) and the stimulus type (*F*_(1,76)_ = 62.215, *p* < 0.01, *η*^2^ = 0.45), but no significant effect of the groups (*F*_(3,76)_ = 0.032, *P* = 0.992 (i.e., >0.05), *η*^2^ = 0.001). There was a significant interaction between the stimulus type × training stage (*F*_(1,76)_ = 30.093, *p* < 0.01, *η*^2^ = 0.284), but no significant interaction between the group × training stage (*F*_(3,76)_ = 0.056, *P* = 0.982 (i.e., >0.05), *η*^2^ = 0.002) or the group × stimulus type (*F*_(3,76)_ = 0.586, *P* = 0.626 (i.e., >0.05), *η*^2^ = 0.023). There was also no significant interaction of three factors (*F*_(3,76)_ = 0.643, *P* = 0.589 (i.e., >0.05), *η*^2^ = 0.025). A paired *t*-test of the last trial of the training session in each group showed no significant difference in SCR to CS+ than to CS− (1/3 group: *t* = 0.237, *P* = 0.815 (i.e., >0.05); 2/3 group: *t* = 0.171, *P* = 0.866 (i.e., >0.05); 3/3 group: *t* = 1.94, *P* = 0.067 (i.e., >0.05); no retrieval group: t = −0.711, *P* = 0.486 (i.e., >0.05). These results showed that extinction training abolished discrimination between the CS+ and CS− in participants of all four groups.

### Test Performance

#### Spontaneous Recovery

**Figure 3:**
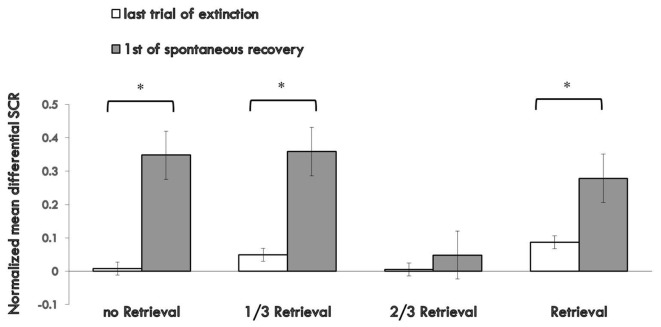
Mean differential SCRs to the last trial of extinction and first trial of spontaneous recovery in each group. The * represent *p* < 0.05. Error bars represent standard errors.

Figure 3 shows the md SCR of the three experimental groups and control group to CSs trials during the spontaneous recovery test (Supplementary Figure S3).

A mixed ANOVA with groups and trials (first trial of spontaneous recovery, last trial of extinction) revealed a significant main effect of the trials (*F*_(1,76)_ = 23.065, *p* < 0.01, *η*^2^ = 0.233) and the groups (*F*_(3,76)_ = 3.329, *p* < 0.05, *η*^2^ = 0.116), but no significant interaction effect between the two (*F*_(3,76)_ = 2.14, *P* = 0.102 (i.e., >0.05), *η*^2^ = 0.078). *Post hoc* analysis showed significant differences between the 2/3 retrieval group and no retrieval group (*p* < 0.05); 2/3 retrieval group and 1/3 retrieval group (*p* < 0.05); and 2/3 retrieval group and whole retrieval group (*p* < 0.05). No differences between other groups were detected.

To test the spontaneous recovery of fear, we compared the md SCR between the first trial of the re-extinction session and the last trial of extinction training in each group. A paired *t*-test showed no significant difference only in the 2/3 retrieval group (*t* = 0.748, *P* = 0.464 (i.e., >0.05), but significant differences were revealed in all other three groups (no retrieval group: *t* = 3.127, *p* < 0.01; 1/3 group: *t* = 2.888, *p* < 0.01; whole retrieval group: *t* = 2.253, *p* < 0.05).

One-way ANOVA between four groups of the first trial of the third day of the SCR to CS+ and CS− revealed a significant main effect of the groups (*F*_(3,76)_ = 3.835, *p* < 0.05, *η*^2^ = 0.131). *Post hoc* analysis showed a significant difference between the 2/3 retrieval group and no retrieval group (*p* < 0.05); 2/3 retrieval group and 1/3 group; and 2/3 retrieval group and whole retrieval group (*p* < 0.05). No differences between other groups were detected.

These results suggested that only the 2/3 repetition retrieval group had no spontaneous recovery of conditioned fear memories 24 h after post-retrieval extinction. Also, the 2/3 repetition retrieval group showed distinct superiority in preventing spontaneous recovery of fear memory compared with the 1/3 retrieval group or whole retrieval group (which is a traditional retrieval parameter). These data suggested that the retrieval ratios of the CS could help to determine the fate of retrieved memory to undergo extinction or reconsolidation.

#### Reinstatement

**Figure 4:**
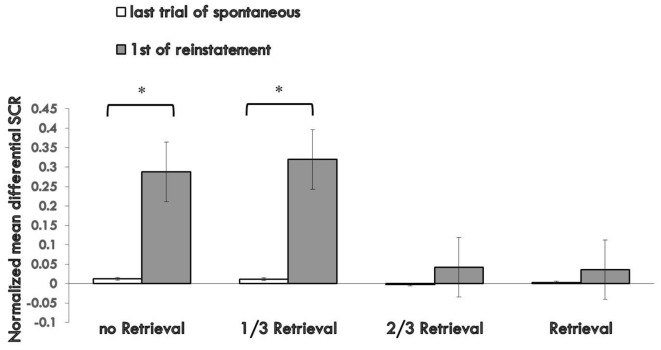
Mean differential SCRs to the last trial of re-extinction and first trial of reinstatement in each group. The * represent *p* < 0.05. Error bars represent standard errors.

To test fear reinstatement, we used the different SCR values between the first trial of reinstatement and last trial of the re-extinction session as the index of the return of fear. Figure 4 shows the md SCR to CSs trials of the four groups during the reinstatement test (Supplementary Figure S4).

A mixed ANOVA with groups and trials (last trial of re-extinction, first trial of reinstatement) revealed a significant main effect of the trials (*F*_(1,76)_ = 13.567, *p* < 0.01, *η*^2^ = 0.151) and significant main effect of the groups (*F*_(3,76)_ = 3.350, *p* < 0.05, *η*^2^ = 0.117), but no significant interaction effect between the two (*F*_(3,76)_ = 2.658, *P* = 0.054 (i.e., >0.05), *η*^2^ = 0.095). *Post hoc* analysis revealed a significant difference between the 3/2 retrieval group and no retrieval group (*p* < 0.05); 2/3 retrieval group and 1/3 retrieval group (*p* < 0.05); and a significant difference between the whole retrieval group and no retrieval group (*p* < 0.05); whole retrieval group and 1/3 retrieval group (*p* < 0.05). No other differences were detected between groups.

A paired *t*-test of the different SCR values between the first trial of the reinstatement session and last trial of re-extinction training in each group showed a significant difference in the no retrieval group (*t* = 2.656, *p* < 0.05) and 1/3 retrieval group (*t* = 3.159, *p* < 0.05) but not in the 2/3 group (*t* = 0.563, *P* = 0.58 (i.e., >0.05)) nor in the whole retrieval group (*t* = 0.446, *P* = 0.661 (i.e., >0.05)).

One-way ANOVA between the differential responses to CS+ and CS− of the four groups of the first trial of reinstatement test showed a significant main effect of the groups (*F*_(3,76)_ = 3.275, *p* < 0.05, *η*^2^ = 0.114). *Post hoc* tests showed the md SCR of the 2/3 retrieval group to be significantly lower than that of the no retrieval group (*p* < 0.05) and 1/3 retrieval group (*p* < 0.05); and that of the whole retrieval group was significantly lower than that of the no retrieval group (*p* < 0.05) and 1/3 retrieval group (*p* < 0.05).

**Figure 5:**
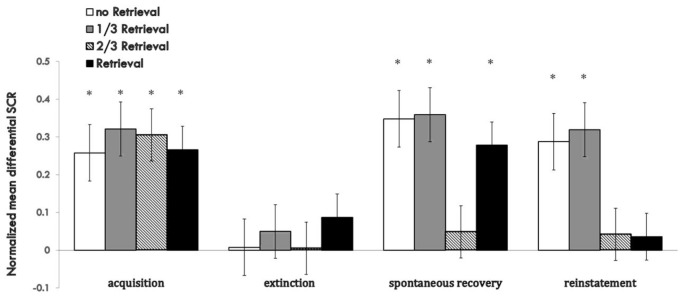
Mean differential SCRs to CS during acquisition, reactivation, extinction, spontaneous recovery test and reinstatement test in each group. *p < 0.05 (between acquisition and extinction, or between extinction and re-extinction within group). Error bars represent standard errors.

The whole performance of the four groups on the index of md SCR during acquisition, extinction and test sessions are shown in Figure 5 (Supplementary Figure S5).

For the index of reinstatement of fear memory, results showed that the 2/3 repetition retrieval group and the whole retrieval group had a distinct effect on preventing relapse, and there were no significant differences between the two. However, the 1/3 repetition retrieval group and no retrieval group failed to prevent fear reinstatement, data that were similar to the results for the spontaneous recovery test. These results inferred that too few similarities were too weak to activate former memories or open the reconsolidation window. A greater proportion of replication to the initial CS as a reminder was needed to achieve a better relapse inhibitory effect. When comparing the relative superiority between the 2/3 retrieval group and whole retrieval group, the latter could only prevent reinstatement and had a poor effect on spontaneous recovery, but the effect of the 2/3 retrieval group was more stable across the two indices of fear return.

## Discussion

We used a basic visual compound CS model as the CS to ascertain whether partial repetition of the compound CSs in conditioning can reactivate memory into reconsolidation. The results showed that the no retrieval group or the 1/3 ratio retrieval group failed to open the memory reconsolidation time window. The 2/3 repetition retrieval group and the whole repetition retrieval group could prevent fear reinstatement, whereas only a 2/3 ratio repetition of the initial compound CS as a reminder could inhibit spontaneous recovery.

Our findings could be interpreted in the context of a reconsolidation hypothesis and add to evidence suggesting the effectiveness of a post-reactivation extinction procedure. If we can “open” the reconsolidation window, then rewriting or updating memory becomes possible. The critical problem is whether the reminder cues are sufficiently valid.

Some researchers have failed to demonstrate the superiority of this paradigm because: (i) the protocol of starting the reconsolidation is invalid; and (ii) subtle changes of the parameter can diminish the difference between retrieval extinction and traditional extinction (Merlo et al., 2014; Sevenster et al., 2014; Nader, 2015). The fate of memory after reactivation lies in multiple possibilities and is dependent upon a series of factors. Memories that are activated may come into the process of reconsolidation or extinction or some period between the two. Only if the retrieved memory meets certain and specific conditions can it go into the reconsolidation process (Fernández et al., 2016a). Traditionally, these boundary conditions include memory age, memory strength and certain conditions terms of retrieval strength (e.g., times and durations of reminder presentation). More recently, the PE has been proposed as a non-invasive index of memory destabilization (Sevenster et al., 2013), whereas some scholars regard it as a necessary (but insufficient) condition for inducing reconsolidation. Hence, it has been suggested that boundary conditions contain two types of factors: (i) a characteristic of acquired memory itself; and (ii) a specific operation process or protocol. These two conditions together determine whether the memory undergoes reconsolidation.

Recently, a new viewpoint suggested that the boundary conditions are not fixed but instead are variable as a result of the interaction between memory features and the characteristics of reminders (Fernández et al., 2016a,b). Hence, boundary conditions should be considered as a “combined” factor and the two aspects should be taken into account when predicting the efficiency of parameters.

In accordance with findings using a complex model of fear stimuli in rats (Jones et al., 2013), we showed that post-retrieval extinction can also be effective in more complex fear memory in humans. This hypothesis also fits with evidence suggesting that retrieval cues which trigger reconsolidation of fear memory are not necessarily a copy of original learning but instead can be flexible, such as abstract cues (Soeter and Kindt, 2015b).

Our results are also consistent with evidence showing the PE to be a boundary condition. Winters et al. (2009) demonstrated that older and stronger object memories which resist impairment can be destabilized by retrieval in the presence of new information. Studies have shown that the PE is a necessary condition that initiates the reconsolidation process (Diaz-Mataix et al., 2013; Sevenster et al., 2013, 2014). New information may produce a need/motivation to update former knowledge frames and acts as a driving factor (Soeter and Kindt, 2015a; Fernández et al., 2016b). However, reconsolidation will no longer be triggered if reactivation contains too much new learning (Sevenster et al., 2014). Increasing evidence suggests that the degree/amount of the PE is a crucial factor that induces reconsolidation rather than new extinction learning or mere retrieval (Kindt et al., 2014; Beckers and Kindt, 2017). Some researchers have found that presentation of multiple reward predictors followed by an absence of reward (extinction) can improve extinction learning, which is interpreted by a larger PE generated than presentation of a single predictor alone (Janak et al., 2012; Furlong et al., 2015). According to the PE theory, the larger the PE, the greater is the associability of the CS, which means the ability to enter into new associations (Holland and Schiffino, 2016).

In our study, results showing that no retrieval or 1/3 repetition retrieval group cannot induce reconsolidation can be interpreted based on the different degree of the PE generated from the four groups. In the acquisition, we built a compound associative learning: CSs–US connection. According to the rate-based model of conditioning, the content of conditioned learning related to compound CSs is related linearly to the reinforcement rate (Gallistel and Gibbon, 2000), which has been shown by some studies (Harris et al., 2012). Hence, in our study, participants acquired one element reinforcement rate (i.e., rA) as being equal to a whole r (rABC) minus the r of other two (rBC). This inferred that the expected US possibility of one element will be 1/3 of the initial US paired to the three-element compound. Hence, appearance of two or all three elements in the retrieval trial can generate more PE by US omission than by only one or no element. In the no retrieval group, there was no reactivation of fear and no PE was generated, which was traditional extinction that formed new learning rather than a change in initial learning. However, in the 1/3 retrieval group, the PE was too small to open the reconsolidation window, which is why this group failed to prevent recovery. A greater proportion of replication of compound CSs as reminders (as shown in 2/3 repetition retrieval and whole repetition retrieval groups), however, would generate a larger PE and consequently result in better destabilization of former memory and, ultimately, disrupt it. These results infer that the memory reconsolidation interference paradigm cannot work if there is insufficient PE at the reactivation period.

In the present study, 2/3 repetition retrieval cues had a better effect in inhibiting spontaneous recovery than those in the whole repetition retrieval group. The result could be interpreted not only according to the PE. A large PE is needed for retrieval but this does not mean that the larger the PE the better is the retrieval (Sevenster et al., 2014). Conversely, not only the change in the CS-US connection but also the change in the CS should be considered in the retrieval period. If the initial CS contains more than one cue it is likely that, in the reactivation, only some of the cues are used, which creates some differences between the CS appearing in retrieval and in acquisition. The change in the CS is a type of novel stimulus that can attract more attention by participants and induce an orientating reflex.

As an important index of the orientating reflex, pupil size has been shown to have a positive correlation with locus coeruleus (LC) neurons (Rajkowski et al., 1993; Gabay et al., 2011). The LC, which is located on the dorsal side of pons, is the main source of norepinephrine (NE) in the brain. Its release of NE is projected widely into cerebral cortices, limbic structures, the thalamus, cerebellum, brainstem and spinal cord (Sara and Bouret, 2012). The noradrenergic system plays an important part in attention shifting and behavioral flexibility (Sara and Bouret, 2012). Studies have revealed that NE is essential for formation of associative fear learning as well as maintenance of long-term memory extinction through the β-receptor signaling system (Janak et al., 2012; Joshua et al., 2014; Furlong et al., 2015). Hence, the LC–NE system, which can be activated if a salient or behaviorally significant stimulus occurs, is very important for the formation or extinction of learning. Whether it can increase or decrease fear conditioning is up to the experimental design. Similar to the way that presentation of a CS can induce two opposite processes, reconsolidation or extinction (Lee et al., 2006), NE activation also has a bidirectional regulatory role in extinction, which can promote consolidation of extinction memory or reconsolidation of fear memory (Wang and Zhu, 2016). It has been shown that the retrieval of memory can activate the noradrenergic system in the amygdala, which enhances reconsolidation and is resistant to extinction (Dębiec et al., 2011). The neural mechanism of the effect of NE on extinction is that activation of the noradrenergic system increases the excitability of neurons and plasticity of synapses between cells (Barth et al., 2007).

Based on these studies, we speculate that 2/3 ratio retrieval with presentation of a changed CS generated a salient and novel stimulus which may increase NE release through the LC–NE system. The activated LC–NE system enhanced the functional connection and plasticity of brain cells. Simultaneously, on account of our experimental design, the activated noradrenergic system promoted memory-reactivation effects and enhanced reconsolidation of fear memory, which facilitated the reconsolidation-interference operation. Hence, the 2/3 ratio retrieval group had a better effect on spontaneous recovery than the whole retrieval group.

Different from some studies showing a consistent effect on testing of spontaneous recovery and reinstatement, we found that the 2/3 retrieval group was superior to the whole retrieval group only for spontaneous recovery and not for reinstatement: they were identical for inhibition of reinstatement. A possible interpretation of this inconsistency is that we tested reinstatement after the spontaneous recovery test (Coelho et al., 2015). Some studies have pointed out that different indices of recovery can interfere with each other (Lonsdorf et al., 2017). Actually, consensus on explorations based on reinstatement in humans is lacking (Haaker et al., 2014). Also, the nature of differences between different types of tests to measure the return of fear is not clear. Hence, further explorations are needed to ascertain the respective mechanism of spontaneous recovery, reinstatement, or other index. However, the present study suggests that exact replication of a CS is not an ideal retrieval cue.

As analyzed above, we demonstrated that after compound CSs, a larger proportion of repetition of the initial CS is needed as a reminder to open the reconsolidation window. That is, sufficient amount of the PE is needed at the retrieval period. Moreover, using a changed CS rather than an initial CS as a reminder can reduce spontaneous recovery, which may be the result of an activated attention system and LC–NE system that promotes NE release. Hence, during the period of reactivation of memory, a change in the CS–US connection (the PE) and the change in the CS may both have effects on reconsolidation of fear memory. Given the potential clinical application, more attention should be paid to the neural mechanism of the PE and the neural mechanism emerging by the change in the CS.

However, there are some limitations of the present study. First, on the test of spontaneous recovery, a statistically significant effect of interaction between the groups and trials is lacking. One possible reason lies in a limited sample size, which needs to be improved in future studies. Second, although the results suggest that the change of CS in reactivation phase may also play a part on destabilizing memory, nevertheless, the effect of PE and CS change cannot be dissociated based on the present paradigm. It will be the main focus of our next study. And last, as we didn’t actually manipulate factor such as NE, the discussion based on the NE release and reconsolidation need more careful inspection, which calls for more exploration in the future.

By demonstrating its effectiveness in more complex models of fear, our results confirm and extend previous findings suggesting that the post-retrieval extinction paradigm may erase fear memory and prevent relapse. Furthermore, if there is more than one CS, using a different ratio of CS as a reminder may have different effects. Too little repetition of a CS failed to reactivate fear memory to undergo reconsolidation. To achieve an optimal and stable effect of inhibiting the return of fear, a moderate-discrepancy CS should be used as a reminder cue to provide an extraneous stimulus to promote reconsolidation. These findings have important clinical implications given that therapy based on impairment of reconsolidation needs careful selection of the reactivation parameters from the many related cues of trauma. Further studies are needed to extend the complex model of fear to cross modalities and explore how the PE and other boundary conditions work in this complex model.

## Author Contributions

XZ and JL designed the experiment and worked on the final version of the manuscript. JL, WC, JC, WW, JJ and LX collected and analyzed the data.

## Conflict of Interest Statement

The authors declare that the research was conducted in the absence of any commercial or financial relationships that could be construed as a potential conflict of interest.
