# Supplementary material for: Moderate Partially Reduplicated Conditioned Stimuli as Retrieval Cue Can Increase Effect on Preventing Relapse of Fear to Compound Stimuli
Source: Front Hum Neurosci. 2017 Nov 30;11:575. doi: 10.3389/fnhum.2017.00575 (PMC5714856; doi:10.3389/fnhum.2017.00575)
Supplement: Supplementary file 1 [file Presentation_1.pptx]

## Slide 1
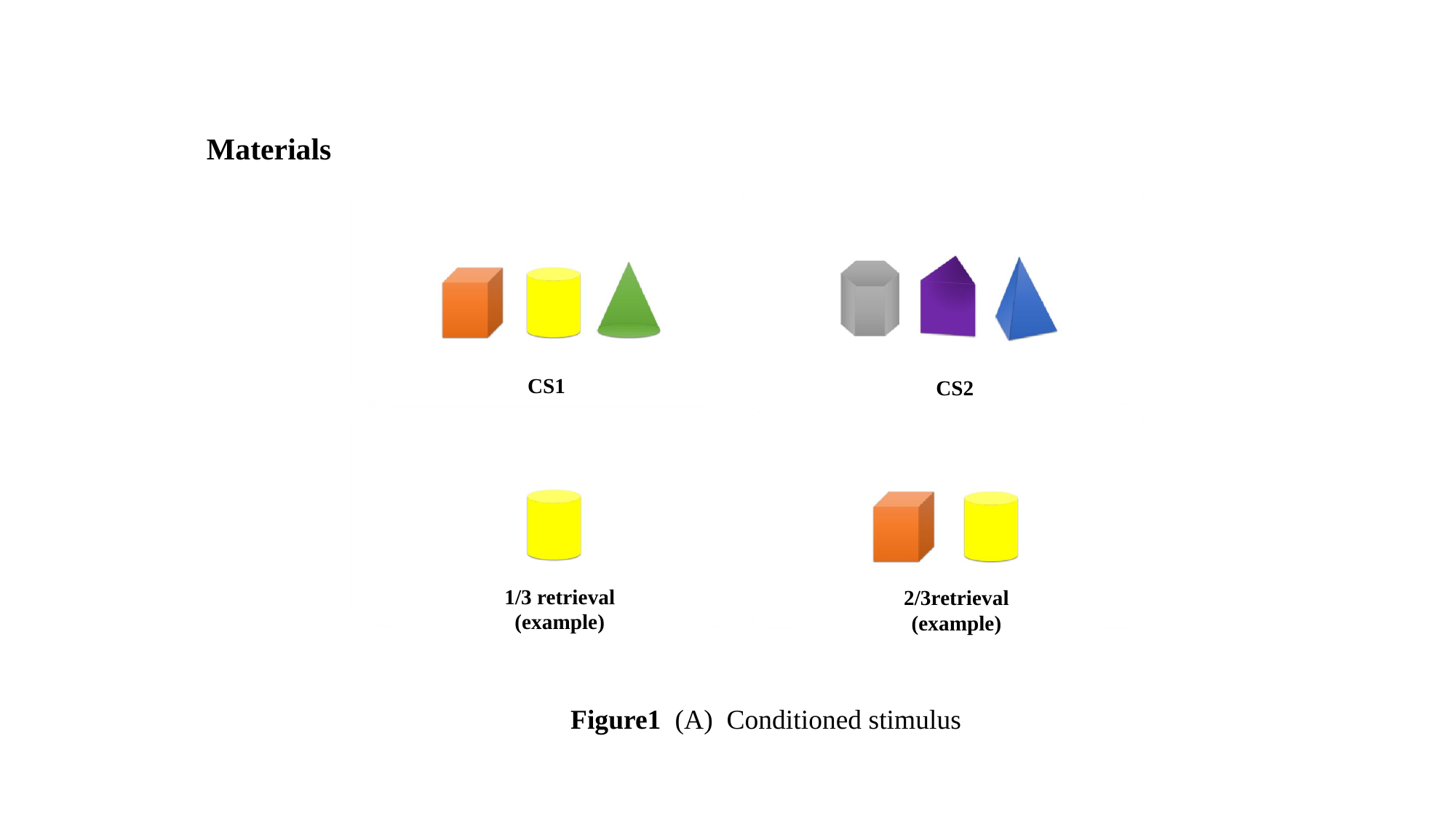

Materials
CS1
CS2
1/3 retrieval (example)
2/3retrieval
(example)
Figure1 (A) Conditioned stimulus

## Slide 2
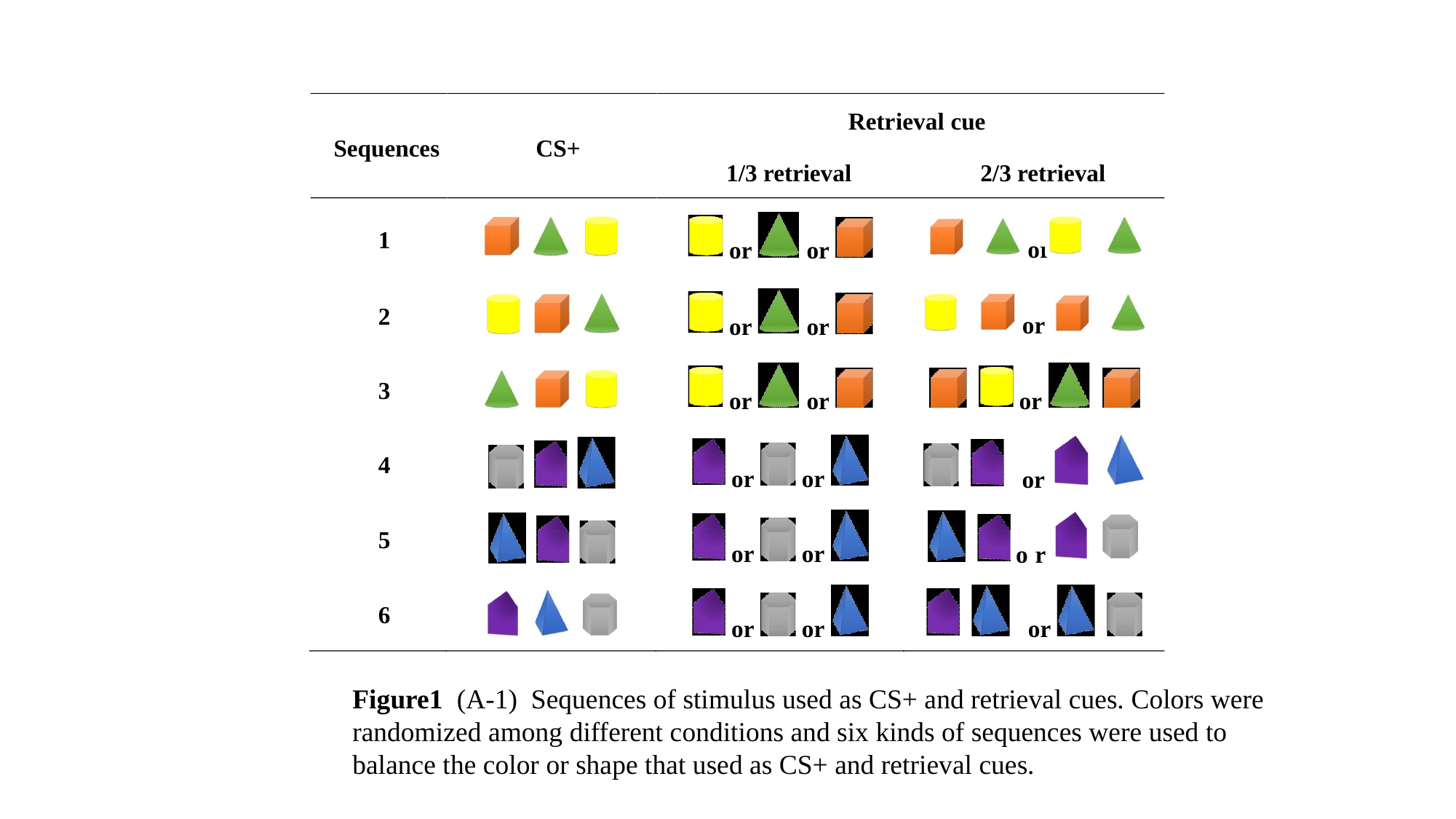

Retr
ieval
cue
Sequences
CS+
1/3 retrieval
2/3 retrieval
1
or
or
or
2
or
or
or
3
or
or
or
4
or
or
or
5
or
or
o
r
6
or
or
or
Figure1 (A-1) Sequences of stimulus used as CS+ and retrieval cues. Colors were randomized among different conditions and six kinds of sequences were used to
balance the color or shape that used as CS+ and retrieval cues.

## Slide 3
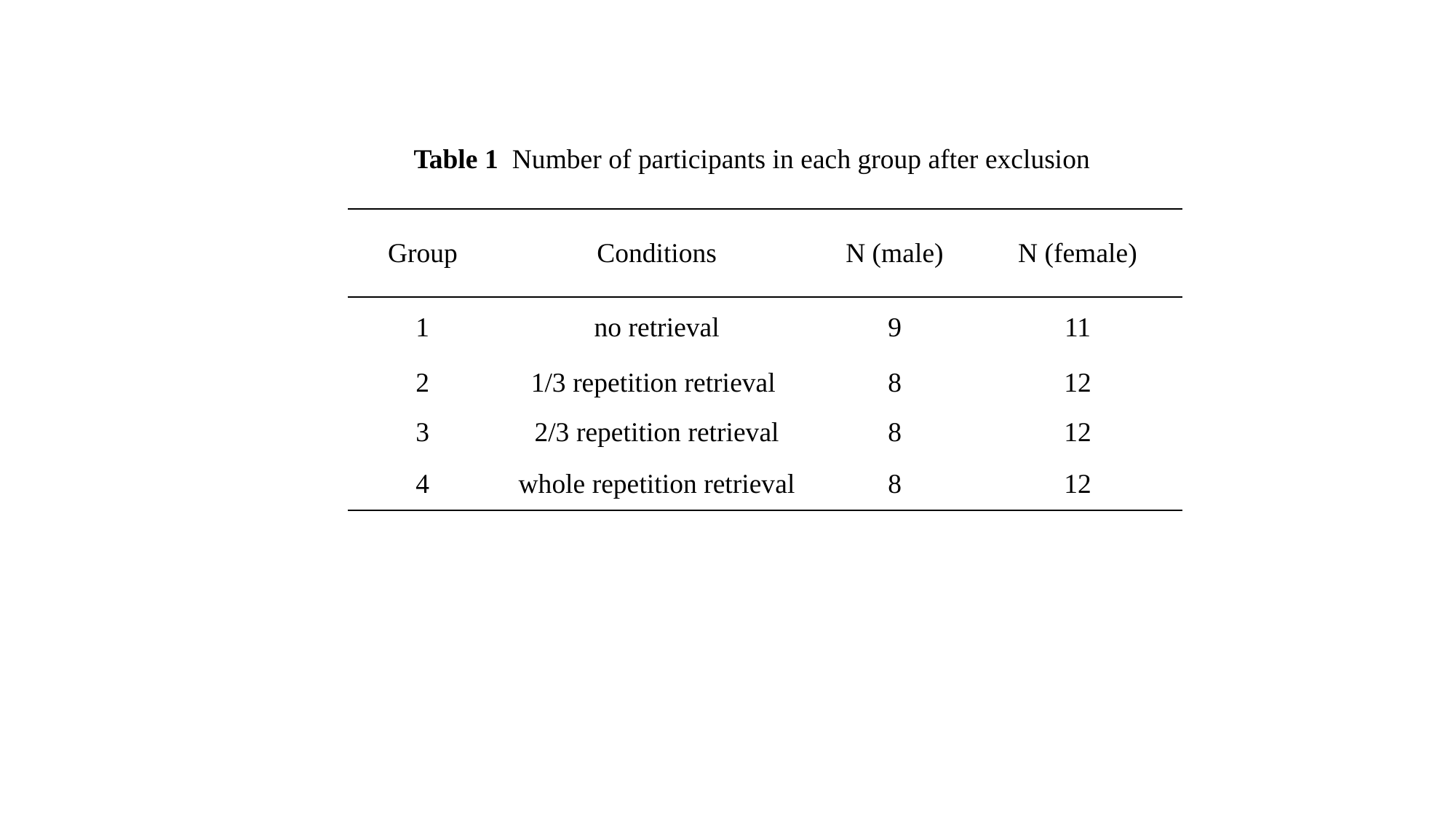

Table 1 Number of participants in each group after exclusion
| Group | Conditions | N (male) | N (female) |
| --- | --- | --- | --- |
| 1 | no retrieval | 9 | 11 |
| 2 | 1/3 repetition retrieval | 8 | 12 |
| 3 | 2/3 repetition retrieval | 8 | 12 |
| 4 | whole repetition retrieval | 8 | 12 |

## Slide 4
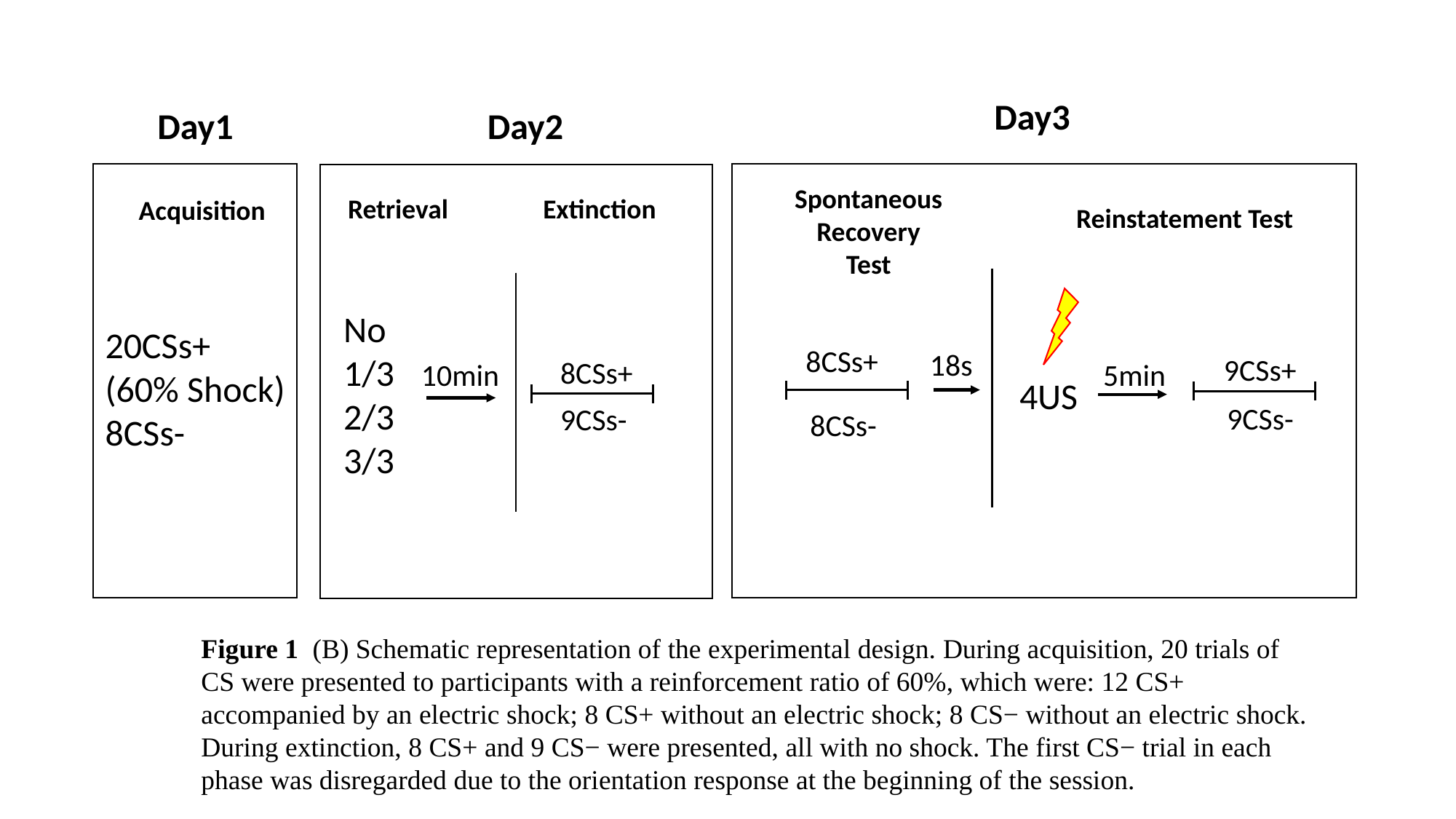

Day3
Day1
Day2
Acquisition
20CSs+
(60% Shock)
8CSs-
Spontaneous
Recovery
Test
Reinstatement Test
8CSs+
18s
9CSs+
5min
4US
9CSs-
8CSs-
Retrieval
Extinction
No
1/3
2/3
3/3
8CSs+
10min
9CSs-
Figure 1 (B) Schematic representation of the experimental design. During acquisition, 20 trials of CS were presented to participants with a reinforcement ratio of 60%, which were: 12 CS+ accompanied by an electric shock; 8 CS+ without an electric shock; 8 CS− without an electric shock. During extinction, 8 CS+ and 9 CS− were presented, all with no shock. The first CS− trial in each phase was disregarded due to the orientation response at the beginning of the session.

## Slide 5
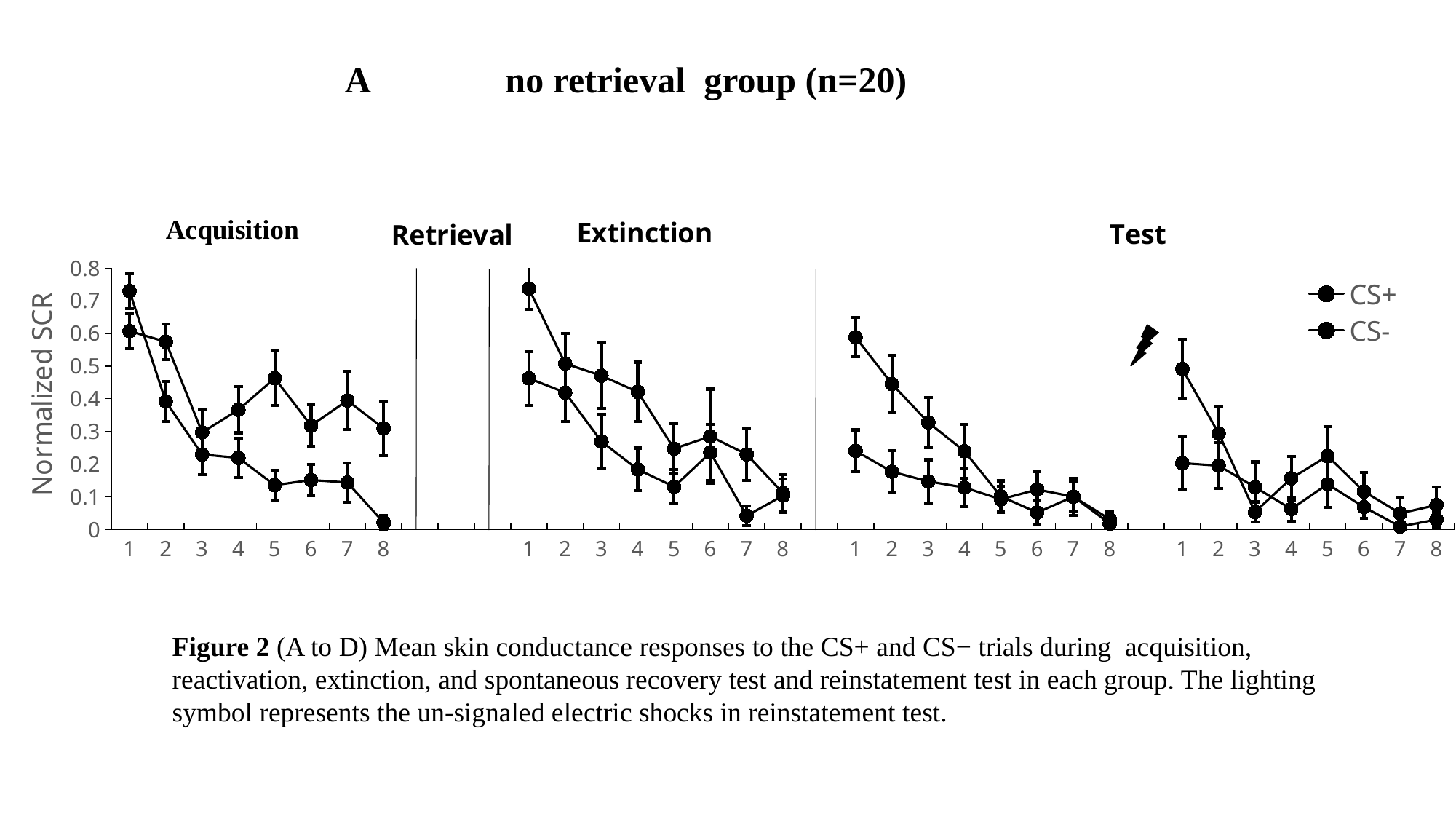

A no retrieval group (n=20)
### Chart
| Category | CS+ | CS- |
|---|---|---|
| 1 | 0.6072748173930991 | 0.7295200752104127 |
| 2 | 0.5745770672811348 | 0.3917014804727713 |
| 3 | 0.29753820403330405 | 0.22989706295576928 |
| 4 | 0.3665502619518264 | 0.2193311043622123 |
| 5 | 0.4629508642930931 | 0.13603230351437745 |
| 6 | 0.3183758900749049 | 0.15170058004640521 |
| 7 | 0.3948793418023443 | 0.14418767951957895 |
| 8 | 0.30992422073012404 | 0.021782123125716594 |
| | None | None |
| | None | None |
| | None | None |
| 1 | 0.7371745595267358 | 0.46287180457388527 |
| 2 | 0.507719067094101 | 0.4192374295143656 |
| 3 | 0.4708906939088747 | 0.26949118137390327 |
| 4 | 0.4213343787846278 | 0.1841342881456213 |
| 5 | 0.24772008891747554 | 0.13127408631644344 |
| 6 | 0.28528795967746134 | 0.2357027513804077 |
| 7 | 0.23018452610306078 | 0.04250745237033034 |
| 8 | 0.11177114774344357 | 0.10413204685723758 |
| | None | None |
| 1 | 0.5888235388789184 | 0.24085289507303012 |
| 2 | 0.4454194533959631 | 0.17703344021954437 |
| 3 | 0.328061805060994 | 0.14768974245150224 |
| 4 | 0.23964814186270228 | 0.12867164220260346 |
| 5 | 0.10180022828362141 | 0.09272742524050538 |
| 6 | 0.052486768990844014 | 0.12266642597496555 |
| 7 | 0.10099212015824106 | 0.10084364031078123 |
| 8 | 0.03203612736159497 | 0.01895395887574076 |
| | None | None |
| 1 | 0.491046394901845 | 0.203386701129643 |
| 2 | 0.29420409922392604 | 0.19554442880020492 |
| 3 | 0.05416344179677307 | 0.1299841887219776 |
| 4 | 0.15682491755836836 | 0.06317111436765674 |
| 5 | 0.22518578420673174 | 0.13910068235950462 |
| 6 | 0.11675555885164912 | 0.0691491078360766 |
| 7 | 0.05 | 0.00961775930846532 |
| 8 | 0.07541424647891318 | 0.031175221669928244 |Figure 2 (A to D) Mean skin conductance responses to the CS+ and CS− trials during acquisition, reactivation, extinction, and spontaneous recovery test and reinstatement test in each group. The lighting symbol represents the un-signaled electric shocks in reinstatement test.

## Slide 6
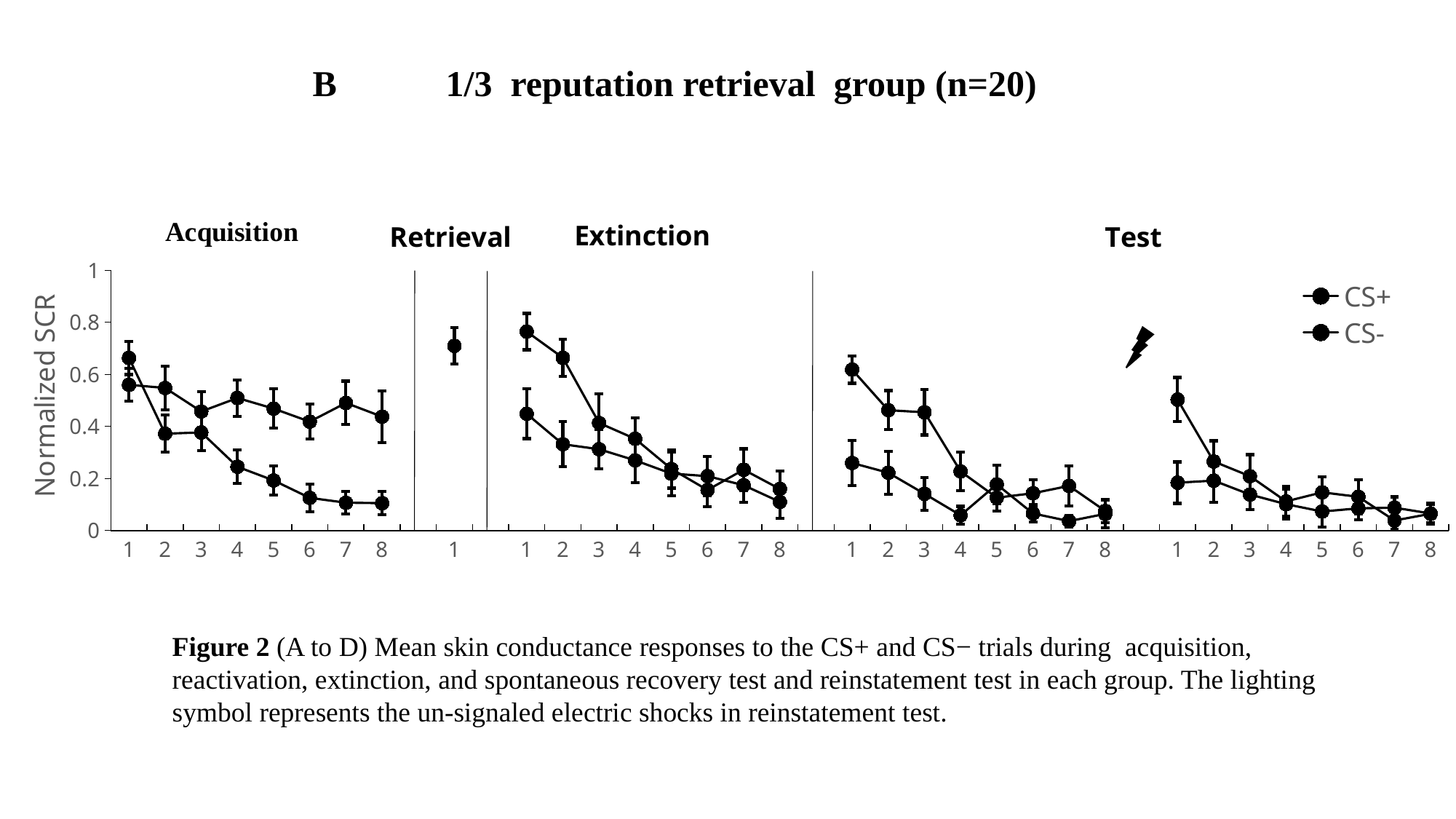

B 1/3 reputation retrieval group (n=20)
### Chart
| Category | CS+ | CS- |
|---|---|---|
| 1 | 0.5599466989839426 | 0.6633532111945948 |
| 2 | 0.5478559788207493 | 0.37199783652508295 |
| 3 | 0.4573028836429569 | 0.3769331714191634 |
| 4 | 0.5094095583019245 | 0.24498943179951102 |
| 5 | 0.4686010461621346 | 0.19234229381781026 |
| 6 | 0.41821482898784634 | 0.1255226557537294 |
| 7 | 0.4903994535525363 | 0.10674302057054134 |
| 8 | 0.43741077367448067 | 0.10510768686734409 |
| | None | None |
| 1 | 0.7097403776778711 | None |
| | None | None |
| 1 | 0.764444456262819 | 0.44850203601180605 |
| 2 | 0.6639437115317376 | 0.3314379207561096 |
| 3 | 0.4132007524579204 | 0.3123709666402771 |
| 4 | 0.35236575431916795 | 0.26958166445249104 |
| 5 | 0.23660909980713263 | 0.21869203670650844 |
| 6 | 0.15570888041121025 | 0.2089493479046136 |
| 7 | 0.23364350625684968 | 0.17382066410612612 |
| 8 | 0.15967479996629108 | 0.110082588407862 |
| | None | None |
| 1 | 0.61832508493748 | 0.2594762333269397 |
| 2 | 0.4625630972304403 | 0.22197500157560096 |
| 3 | 0.4543029558632627 | 0.1409445680306532 |
| 4 | 0.22712390194447035 | 0.05851082362035341 |
| 5 | 0.126119938183699 | 0.17711747079164147 |
| 6 | 0.14312713349407954 | 0.06554927737838737 |
| 7 | 0.1713434173074871 | 0.036121350965759594 |
| 8 | 0.075063388559772 | 0.06380981540565814 |
| | None | None |
| 1 | 0.5030106127024678 | 0.18362554585130725 |
| 2 | 0.26518472838749785 | 0.19180442095251218 |
| 3 | 0.20878626288712393 | 0.1382630375326694 |
| 4 | 0.11189790422581133 | 0.1012234715213561 |
| 5 | 0.14657994364012336 | 0.07288859862496207 |
| 6 | 0.1294408931013878 | 0.08508522401060027 |
| 7 | 0.03838050151826293 | 0.08757526703683023 |
| 8 | 0.0643729827587283 | 0.06527792167696626 |Figure 2 (A to D) Mean skin conductance responses to the CS+ and CS− trials during acquisition, reactivation, extinction, and spontaneous recovery test and reinstatement test in each group. The lighting symbol represents the un-signaled electric shocks in reinstatement test.

## Slide 7
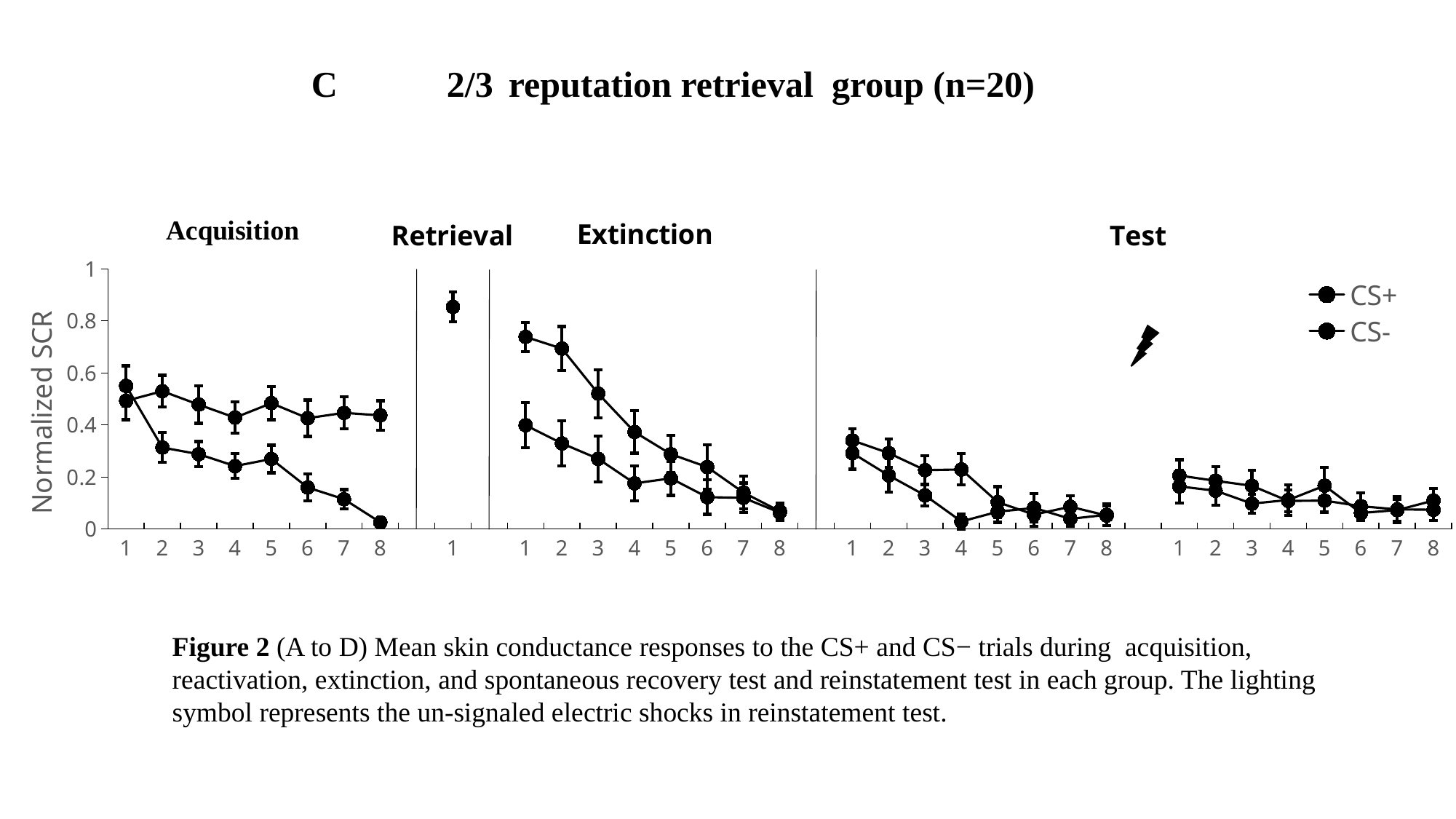

C 2/3 reputation retrieval group (n=20)
### Chart
| Category | CS+ | CS- |
|---|---|---|
| 1 | 0.4927994540095765 | 0.550106981278909 |
| 2 | 0.5297083734375367 | 0.31302337921999757 |
| 3 | 0.47809287248656585 | 0.28751326971634683 |
| 4 | 0.42802631985082346 | 0.2417908051890139 |
| 5 | 0.483671446800665 | 0.26899515416138764 |
| 6 | 0.42574917274992413 | 0.15950441795455889 |
| 7 | 0.44629470068037974 | 0.11454212931641927 |
| 8 | 0.4365956103796836 | 0.025672347427767868 |
| | None | None |
| 1 | 0.8538245606182251 | None |
| | None | None |
| 1 | 0.7384397698441715 | 0.3987447823026538 |
| 2 | 0.6934775572812101 | 0.32898097828779094 |
| 3 | 0.5203180677438038 | 0.2696631562063743 |
| 4 | 0.372792582385788 | 0.1754605107488485 |
| 5 | 0.287689842050357 | 0.19459252692596588 |
| 6 | 0.23803756659893627 | 0.12240671692302038 |
| 7 | 0.140058401750596 | 0.11966207595103832 |
| 8 | 0.06838804005507763 | 0.0632167050068052 |
| | None | None |
| 1 | 0.3403043045446282 | 0.29168620414488283 |
| 2 | 0.2917515299129117 | 0.20570434868902163 |
| 3 | 0.22624748031281858 | 0.12915974882915732 |
| 4 | 0.22865055265430895 | 0.02892836358655506 |
| 5 | 0.10316106584862379 | 0.06626611152589525 |
| 6 | 0.05541594737442177 | 0.08106922102426377 |
| 7 | 0.08554795728538975 | 0.03909545556374964 |
| 8 | 0.05175666680831466 | 0.054377243264530396 |
| | None | None |
| 1 | 0.20541631418380324 | 0.16357596318474016 |
| 2 | 0.1851750932914361 | 0.14708531823852172 |
| 3 | 0.1659087649509811 | 0.0974742221667392 |
| 4 | 0.10779537014908062 | 0.1114548693305573 |
| 5 | 0.10913339521276395 | 0.16642014053154497 |
| 6 | 0.08762138418759044 | 0.06152541514480124 |
| 7 | 0.07527248380041963 | 0.07255018991301013 |
| 8 | 0.07402042766649827 | 0.10922626805380212 |Figure 2 (A to D) Mean skin conductance responses to the CS+ and CS− trials during acquisition, reactivation, extinction, and spontaneous recovery test and reinstatement test in each group. The lighting symbol represents the un-signaled electric shocks in reinstatement test.

## Slide 8
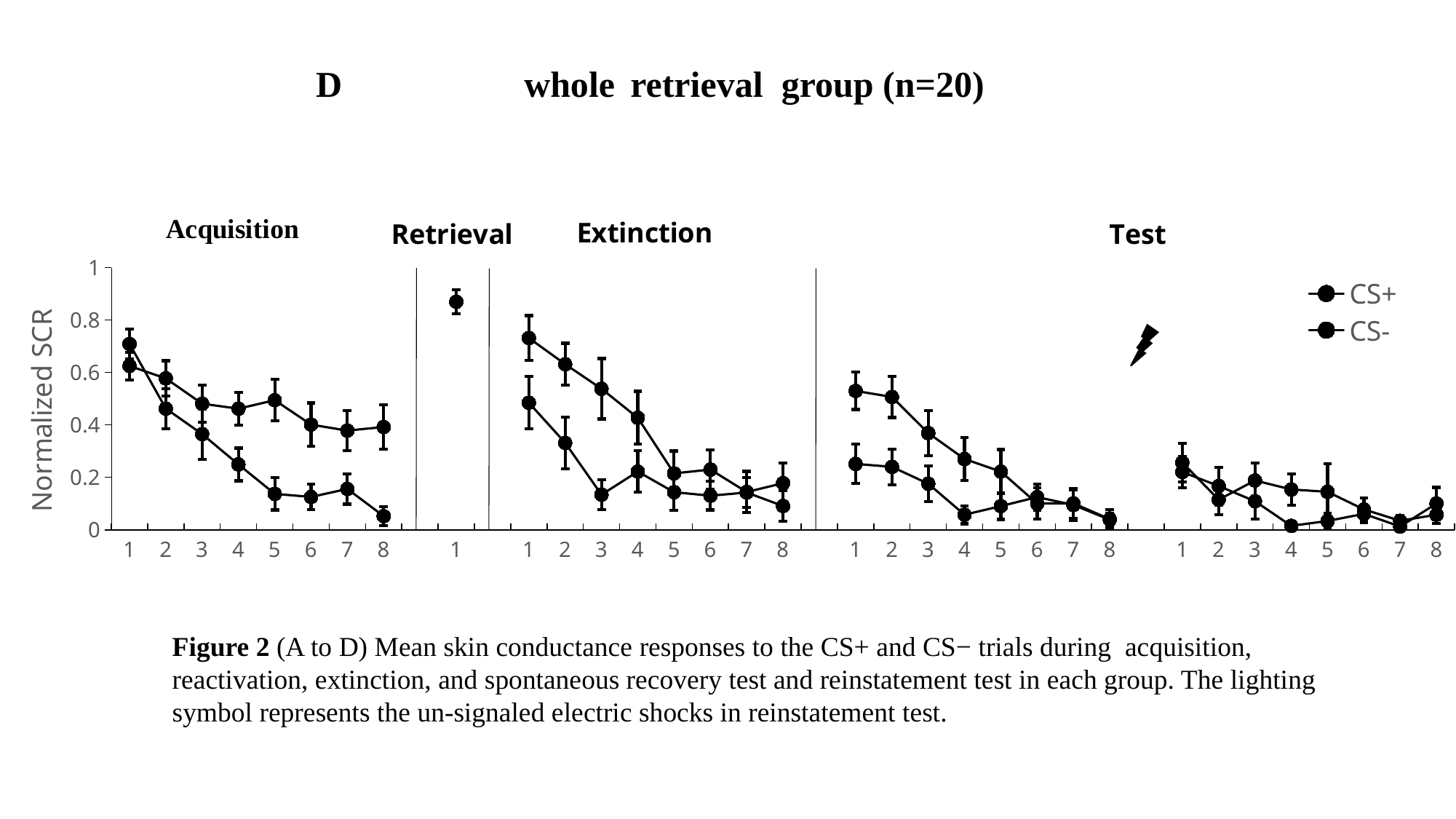

D whole retrieval group (n=20)
### Chart
| Category | CS+ | CS- |
|---|---|---|
| 1 | 0.6254037394443656 | 0.709444244017427 |
| 2 | 0.5779106954779009 | 0.4628906799505338 |
| 3 | 0.4814802722477064 | 0.36549105199057436 |
| 4 | 0.4624779681331372 | 0.2498177188694918 |
| 5 | 0.4950001933941116 | 0.1377334965874922 |
| 6 | 0.4017695019869241 | 0.12592265936565986 |
| 7 | 0.37876315806625466 | 0.1563443746190297 |
| 8 | 0.392472987610055 | 0.052803450363258286 |
| | None | None |
| 1 | 0.8700390043472694 | None |
| | None | None |
| 1 | 0.7320937326703231 | 0.48524802463223227 |
| 2 | 0.6319073682445969 | 0.33203209390390176 |
| 3 | 0.5384078829363681 | 0.13467944173341262 |
| 4 | 0.4275742392620236 | 0.2226377681119917 |
| 5 | 0.21569354377340288 | 0.14408551409830742 |
| 6 | 0.2302191266568736 | 0.13073462869305955 |
| 7 | 0.14479050946652852 | 0.1427483008730896 |
| 8 | 0.17832782330148766 | 0.0913929064177047 |
| | None | None |
| 1 | 0.530318190534992 | 0.2519809235680107 |
| 2 | 0.5069982145172139 | 0.2406522924404635 |
| 3 | 0.36936391949195263 | 0.17638496910563334 |
| 4 | 0.27063025670729673 | 0.05805653704575862 |
| 5 | 0.22266634357282739 | 0.09095811455263278 |
| 6 | 0.10091421737903346 | 0.12544651028087941 |
| 7 | 0.1015631728431368 | 0.09527422720729148 |
| 8 | 0.04132809720950646 | 0.03923733994706737 |
| | None | None |
| 1 | 0.25715324023097896 | 0.2211928221568372 |
| 2 | 0.11543782314818658 | 0.1675045634190387 |
| 3 | 0.18873444520352045 | 0.10971691651949658 |
| 4 | 0.15439917872120026 | 0.015729687756693537 |
| 5 | 0.1454308251373457 | 0.034539804150621425 |
| 6 | 0.07843992703048572 | 0.06128671184340456 |
| 7 | 0.035102764578942 | 0.013111860517013168 |
| 8 | 0.05827687750028286 | 0.1009629994858607 |Figure 2 (A to D) Mean skin conductance responses to the CS+ and CS− trials during acquisition, reactivation, extinction, and spontaneous recovery test and reinstatement test in each group. The lighting symbol represents the un-signaled electric shocks in reinstatement test.

## Slide 9
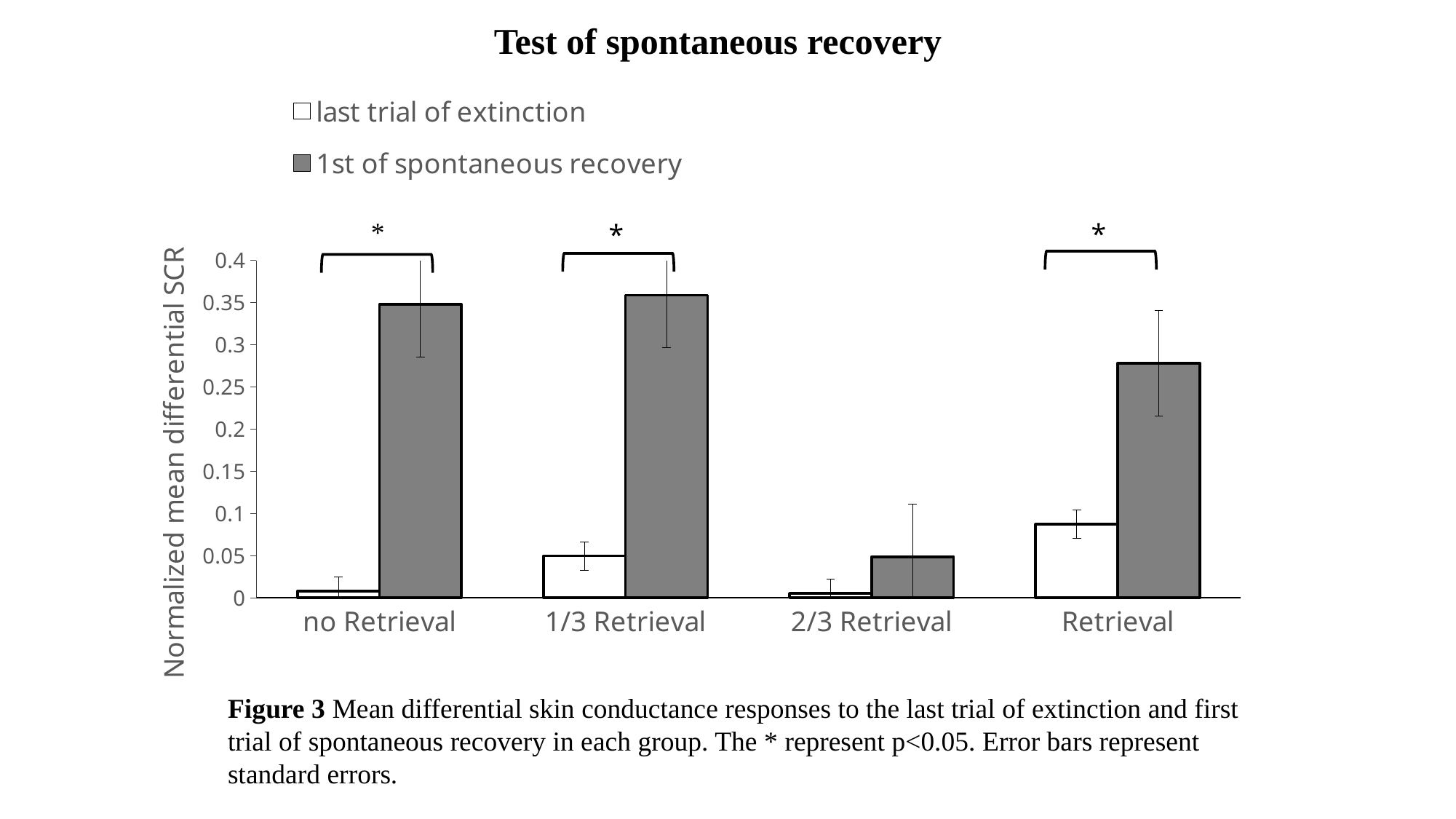

Test of spontaneous recovery
### Chart
| Category | last trial of extinction | 1st of spontaneous recovery |
|---|---|---|
| no Retrieval | 0.007639100886206002 | 0.34797064380588827 |
| 1/3 Retrieval | 0.04959221155842908 | 0.35884885161054025 |
| 2/3 Retrieval | 0.005171335048272446 | 0.04861810039974539 |
| Retrieval | 0.08693491688378299 | 0.2783372669669812 |Figure 3 Mean differential skin conductance responses to the last trial of extinction and first trial of spontaneous recovery in each group. The * represent p<0.05. Error bars represent
standard errors.

## Slide 10
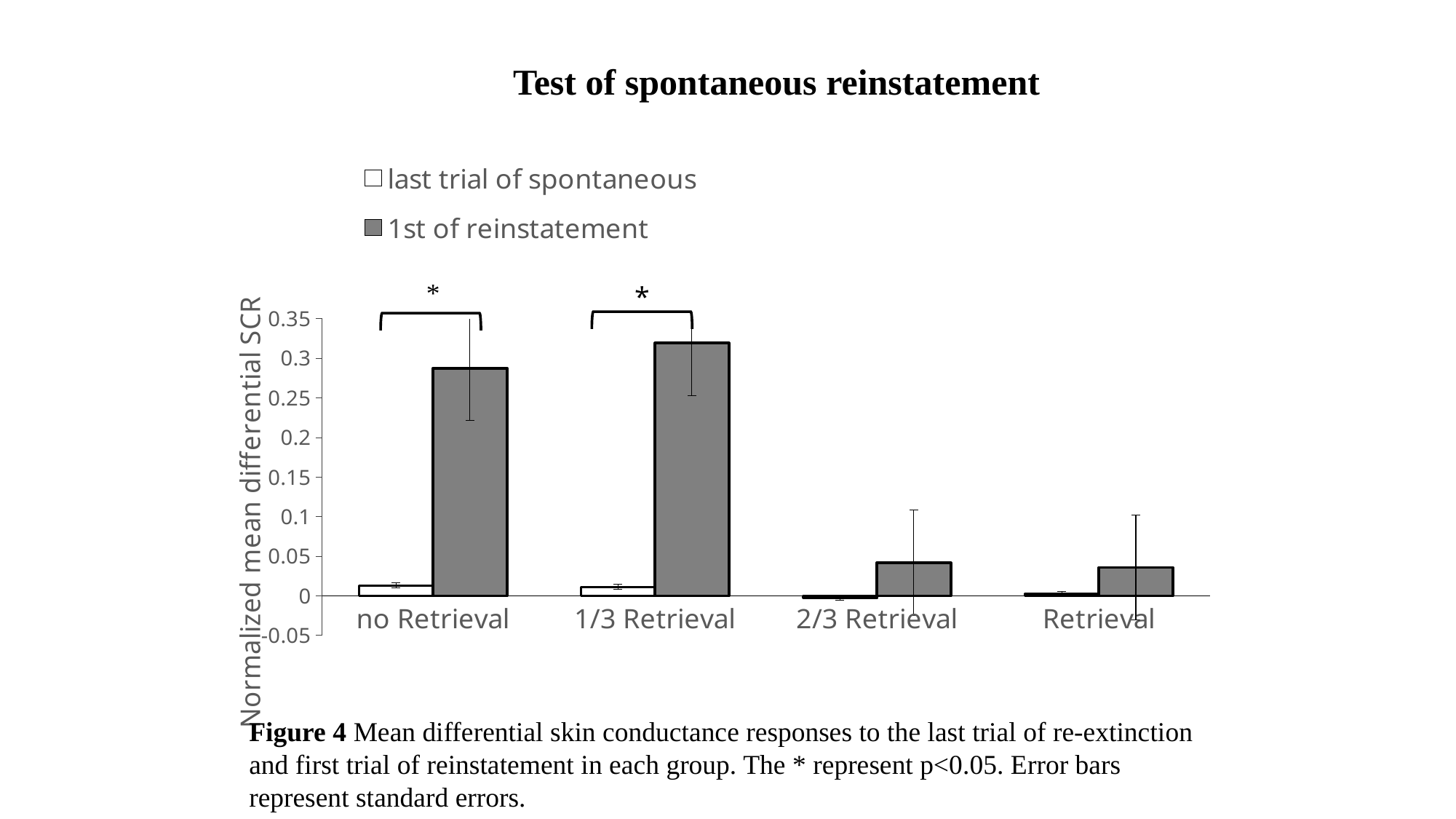

Test of spontaneous reinstatement
### Chart
| Category | last trial of spontaneous | 1st of reinstatement |
|---|---|---|
| no Retrieval | 0.013082168485854206 | 0.2876596937722019 |
| 1/3 Retrieval | 0.011253573154113865 | 0.3193850668511605 |
| 2/3 Retrieval | -0.002620576456215734 | 0.04184035099906307 |
| Retrieval | 0.002090757262439083 | 0.035960418074141784 |Figure 4 Mean differential skin conductance responses to the last trial of re-extinction and first trial of reinstatement in each group. The * represent p<0.05. Error bars represent standard errors.

## Slide 11
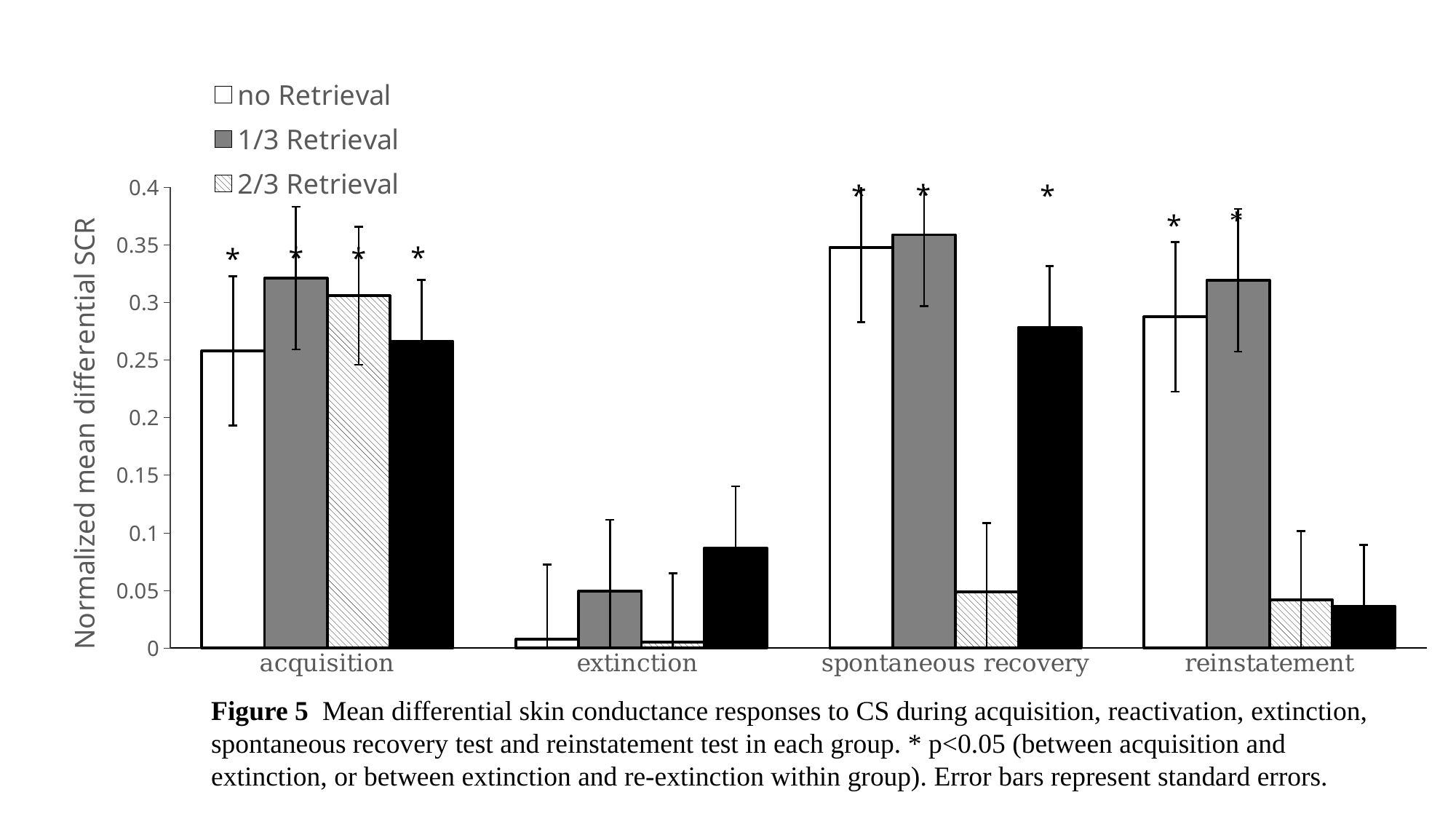

### Chart
| Category | no Retrieval | 1/3 Retrieval | 2/3 Retrieval | Retrieval |
|---|---|---|---|---|
| acquisition | 0.258106907673597 | 0.3212276113418932 | 0.30589922043762974 | 0.26625274080199335 |
| extinction | 0.007639100886206002 | 0.04959221155842908 | 0.005171335048272446 | 0.08693491688378299 |
| spontaneous recovery | 0.34797064380588827 | 0.35884885161054025 | 0.04861810039974539 | 0.2783372669669812 |
| reinstatement | 0.2876596937722019 | 0.3193850668511605 | 0.04184035099906307 | 0.035960418074141784 |Figure 5 Mean differential skin conductance responses to CS during acquisition, reactivation, extinction, spontaneous recovery test and reinstatement test in each group. * p<0.05 (between acquisition and extinction, or between extinction and re-extinction within group). Error bars represent standard errors.
